# Supplementary material for: Hypoglycemia prevention practice and associated factors among diabetes mellitus patients in Ethiopia: Systematic review and meta-analyssis
Source: PLoS One. 2022 Nov 1;17(11):e0275786. doi: 10.1371/journal.pone.0275786 (PMC9624414; doi:10.1371/journal.pone.0275786)
Supplement: S1 Fig — (DOCX) [file pone.0275786.s002.docx]

**Identification of studies via databases and registers**

Records removed *before screening*:

Duplicate records removed (n =8)

Records marked as ineligible by automation tools (n = 100)

Records removed for other reasons (n = 10)

Records identified from*:

Databases (n =149)

Registers (n =10)

**Identification**

Records screened

(n = 41)

Records excluded**

(n =4)

Reports sought for retrieval

(n = 37)

Reports not retrieved

(n =7)

**Screening**

Reports assessed for eligibility

(n = 30)

Reports excluded:

Reason 1 (n =7)

Reason 2 (n = 4)

Reason 3 (n =2)

etc.

s

Studies included in review

(n = 17)

Reports of included studies

(n =12)

**Included**

**Figure 1.** PRISMA diagram of selecting and including studies for systematic review and meta-analysis for the prevention practice of hypoglycaemia and associated factors among diabetic patients in Ethiopia, 2021

*Consider, if feasible to do so, reporting the number of records identified from each database or register searched (rather than the total number across all databases/registers).

**If automation tools were used, indicate how many records were excluded by a human and how many were excluded by automation tools.

*From:*  Page MJ, McKenzie JE, Bossuyt PM, Boutron I, Hoffmann TC, Mulrow CD, et al. The PRISMA 2020 statement: an updated guideline for reporting systematic reviews. BMJ 2021; 372:n71. doi: 10.1136/bmj.n71

For more information, visit: <http://www.prisma-statement.org/>
