# Supplementary material for: Hypoglycemia prevention practice and associated factors among diabetes mellitus patients in Ethiopia: Systematic review and meta-analyssis
Source: PLoS One. 2022 Nov 1;17(11):e0275786. doi: 10.1371/journal.pone.0275786 (PMC9624414; doi:10.1371/journal.pone.0275786)
Supplement: Attachment — Submitted filename: Comments to the Author by Kirubel Eshetu.docx [file pone.0275786.s003.docx]

Comments to the Authors by Kirubel Eshetu

This manuscript addresses an interesting and relevant topic,and it is very important to work for Ethiopia. We know that we have very limited data. Therefore, this manuscript helps to advance the paper in the country.

**Comment to the authors**

1. Grammar errors need to be corrected. The manuscript presented in an intelligible fashion and written in standard English.
2. Reduce the volume of the abstraction and introduction and write the story chronologically. Currently, it is haphazard.
3. On conclusion focus only with your findings

**Questions to the authors**

1. How did you assess pooled prevalence?
2. what type of study designs you include in this review?
3. which study tool was used to assess review outcome?
4. How you reduce the bias of this review?
